# The Manufacture of Xeno- and Feeder-Free Clinical-Grade Human Embryonic Stem Cell Lines: First Step for Cell Therapy

**DOI:** 10.3390/ijms232012500

**Published:** 2022-10-18

**Authors:** Tereza Souralova, Daniela Rehakova, Michal Jeseta, Lenka Tesarova, Jindrich Beranek, Pavel Ventruba, Ales Hampl, Irena Koutna

**Affiliations:** 1 Cell and Tissue Engineering Facility, International Clinical Research Center, St. Anne’s University Hospital, Pekarska 664/53, 60200 Brno, Czech Republic; 2 Department of Histology and Embryology, Faculty of Medicine, Masaryk University, Kamenice 5, 62500 Brno, Czech Republic; 3 Department of Experimental Biology, Faculty of Science, Masaryk University, Kamenice 5, 62500 Brno, Czech Republic; 4 Center of Assisted Reproduction, Department of Gynecology and Obstetrics, University Hospital Brno, Masaryk University, Jihlavska 20, 62500 Brno, Czech Republic; 5 Cell and Tissue Regeneration, International Clinical Research Center, St. Anne’s University Hospital, Pekarska 664/53, 60200 Brno, Czech Republic

**Keywords:** hESC, clinical grade, cell therapy, clean rooms, pluripotent stem cells

## Abstract

Human embryonic stem cells (hESCs) are increasingly used in clinical trials as they can change the outcome of treatment for many human diseases. They are used as a starting material for further differentiation into specific cell types and to achieve the desirable result of the cell therapy; thus, the quality of hESCs has to be taken into account. Therefore, current good manufacturing practice (cGMP) has to be implemented in the transport of embryos, derivation of inner cell mass to xeno-free, feeder-free and defined hESC culture, and cell freezing. The in-depth characterization of hESC lines focused on safety, pluripotency, differentiation potential and genetic background has to complement this process. In this paper, we show the derivation of three clinical-grade hESC lines, MUCG01, MUCG02, and MUCG03, following these criteria. We developed and validated the system for the manufacture of xeno-free and feeder-free clinical-grade hESC lines that present high-quality starting material suitable for cell therapy according to cGMP.

## 1. Introduction

Human embryonic stem cells (hESCs) have a unique ability to differentiate into any cell type in the human body and proliferate indefinitely [1]. This ability makes them excellent starting material for the manufacture of a high number of desired specific cell types, e.g., retinal pigment epithelium cells, dopaminergic neurons, or hepatic cells [2,3,4]. Despite these advantages, there is still ethical controversy when a human blastocyst is used for hESC derivation; therefore, the derivation of hESCs is forbidden in some countries. [5,6]. In order to ensure that the ethical aspects of derivation are met, unsuitable or supernumerary human blastocysts for fertility treatments provided with informed consent are used.

Cell therapies involving hESC have shown very promising results for the treatment of certain diseases, especially age-related macular degeneration, and confirmed safety of hESC-derived cells for humans [7,8,9,10]. These results stand behind the rise of cell therapies using hESCs, as their number increased from 11 trials in 2015, followed by 32 trials in 2019 to 47 trials in 2021 [11,12,13,14]. However, the prospective clinical use of hESC-derived cells needs to be further examined from the point of their safety, quality, functionality, and efficacy.

Current good manufacturing practices (cGMP) are guidelines set in place to ensure the proper design, monitoring, and controlling of manufacturing processes, facilities, and operations. These guidelines are defined by regulatory authority, e.g., the U.S. Food and Drug Administration (FDA) for the United States of America (USA) and the European Medicines Agency (EMA) for the European Union (EU) [15,16,17]. Regarding EMA cGMP guidelines, clinical-grade hESC lines have to be established in validated clean rooms with validated equipment [15,18]. The environment monitoring must ensure that the culture is not compromised with bacteria, fungi, or viruses [19]. Whole manufacture and quality control have to be executed according to standard operating procedures (SOPs) and have to be recorded in accordance with principles of controlled documentation. Personnel has to be regularly trained, especially for principles of aseptic manufacture, SOPs concerning manufacture and quality control (QC), and gowning [15,20].

The characterization of hESCs focuses mainly on their pluripotency, differentiation potential, identity establishment, and safety. Differentiation potential, i.e., ability to differentiate into germ layers—endoderm, mesoderm, and ectoderm—differ amongst hESC cell lines [21,22,23]. Some lines fail to differentiate into one germ layer, and therefore it is necessary to evaluate their differentiation potential to distinguish the suitability of certain clinical-grade hESC lines for specific applications. The safety of clinical-grade hESCs lies in genetic background screening—karyotype establishment, cancer-predisposition testing, and whole genome sequencing are suitable methods for this purpose [19,20]. Taken together, the characterization of hESCs should provide information that helps to select a hESC line suitable for further applications of hESC-derived cells in cell therapy.

The previously published cGMP system for the establishment of clinical-grade hESCs used cGMP human feeders for the derivation of hESCs [18,24,25,26]. Although the establishment of cGMP human feeders follows strict rules and human feeders should not be antigenic, they are undefined and heterogeneous by their very nature. Fortunately, various defined xeno-free cGMP substrates can be used instead of feeder cells, e.g., laminin 521 and vitronectin [27]. The defined xeno-free cGMP culture media, freezing media and serum replacements are also available on the market. These defined cGMP products, together with the hypoxia that supports the pluripotent state of hESCs, are the basis of a high-quality clinical-grade hESC culture [17,28,29]. 

In this paper, we describe the cGMP system of the hESC manufacturing with the use of cGMP-defined xeno-free laminin 521 for hESC derivation and expansion together with the quality control requirements concerning the sterility, safety, and characterization of clinical-grade hESCs for further clinical use.

## 2. Results

### 2.1. Development of a System for the Manufacture and QC of Xeno-Free, Feeder-Free Clinical-Grade hESC Lines According to cGMP

To derive clinical-grade hESCs in an appropriate environment, clean rooms of Cell and Tissue Engineering Facility (CTEF) at St. Anne’s University Hospital Brno established in the Czech Republic were used. Clinical-grade hESCs were produced in an A-grade environment in a B-grade room, with entry via a C-grade room.

Before the derivation itself, the extensive testing of suitable cGMP material and procedures took place. The material was chosen based on the quality, involving primarily cGMP, IVF, and IVD products. We improved our techniques, including derivation, on 4 research-grade lines with the same settings as for the clinical-grade hESCs. Moreover, we prepared SOPs and record forms for the manufacture and QC according to the cGMP requirements for advanced therapy medicinal products (ATMPs) [15]. The personnel were trained during the testing and the equipment of the clean rooms were tailored to the purpose.

**Figure 1:**
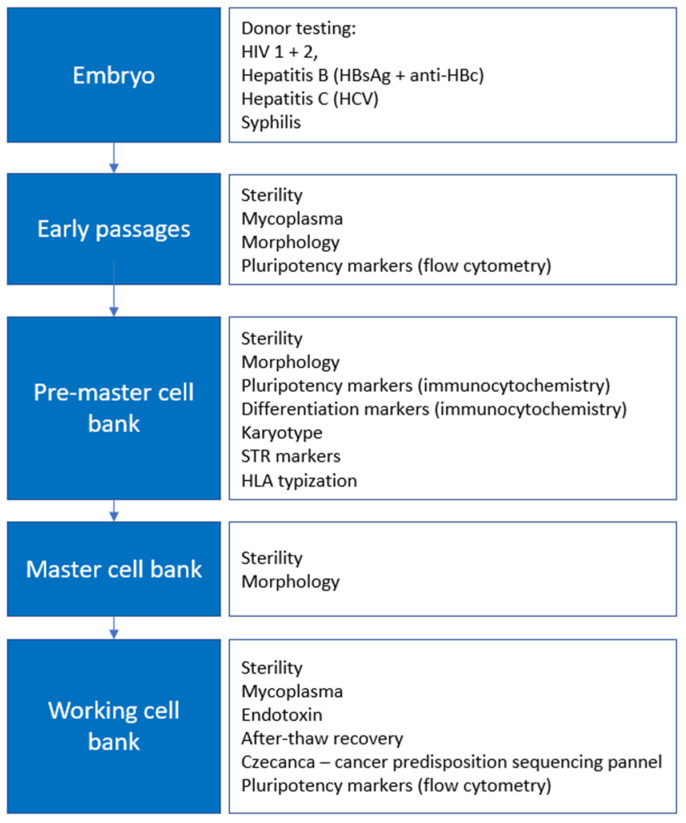
Manufacture scheme: banking of pre-master cell bank, master cell bank, and working cell bank.

The manufacture of clinical-grade hESC lines is divided into several phases: derivation, early expansion into pre-master bank, master cell bank, and expansion into working cell bank that represents the final product (see Figure 1). Each phase is accompanied by the specific QC testing, although the safety is the main specification examined during the entire manufacturing process.

### 2.2. Xeno-Free and Feeder-Free Derivation and Culture of Clinical-Grade hESC Lines According to cGMP

Three clinical-grade lines, MUCG01, MUCG02, and MUCG03, were established from 38 embryos in total (see Table 1), which resulted in a 7.9% success rate. The 4 individual derivations were performed with 8–10 embryos per derivation.

E-cadherin with cGMP laminin 521 was used to support the attachment of the derived inner cell mass. Similarly, human serum albumin and ROCK inhibitor were added to the medium to improve the success of the derivation (see Table 2). Mechanical derivation using IVF micropipettes was performed in order to avoid xeno-free products.

**Figure 2:**
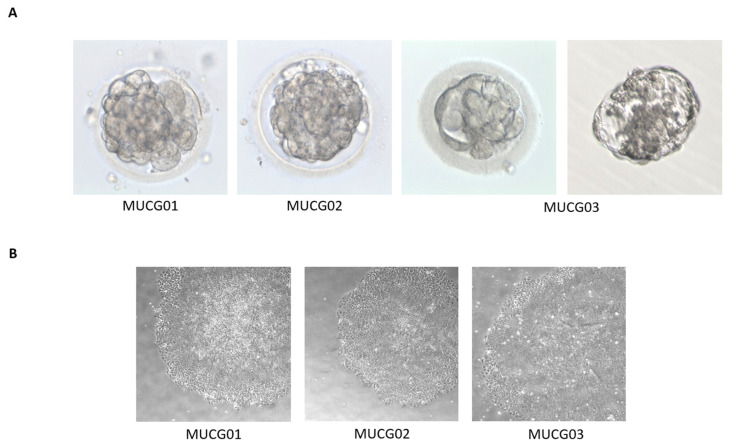
(**A**) Blastocysts used for the derivation of clinical-grade hESC lines MUCG01, MUCG02 and MUCG03. Blastocyst for the derivation of MUCG03 was cultured for two days before transport; the first picture is after the thawing and the second picture before the transport. (**B**) The morphology of clinical-grade hESC line MUCG01 was captured in p0, MUCG02 in p0, and MUCG03 in p3.

Blastocysts used for the derivation of MUCG01, MUCG02, and MUCG03 showed no abnormalities, as shown in Figure 2A. The blastocyst for the derivation of MUCG03 was cultured for two days before transport. The morphology of cells in the outgrowth (p0) for MUCG01 and MUCG02, and the morphology of MUCG03 in p3 matched the morphology of hESCs (see Figure 2B). Colonies were flat with bright edges, and the cells in the middle of the colony were small and round with large nucleus and visible nucleoli. The cells on the edges were slightly bigger.

The culture of clinical-grade hESCs was performed in clean rooms. All operations were executed by trained personnel according to SOPs and recorded according to the controlled documentation. The cells were passaged mechanically by needle for the first three passages and then non-enzymatically in clumps by EDTA (for details see Table 3).

### 2.3. Characterization of the Clinical-Grade hESC Lines

**Figure 3:**
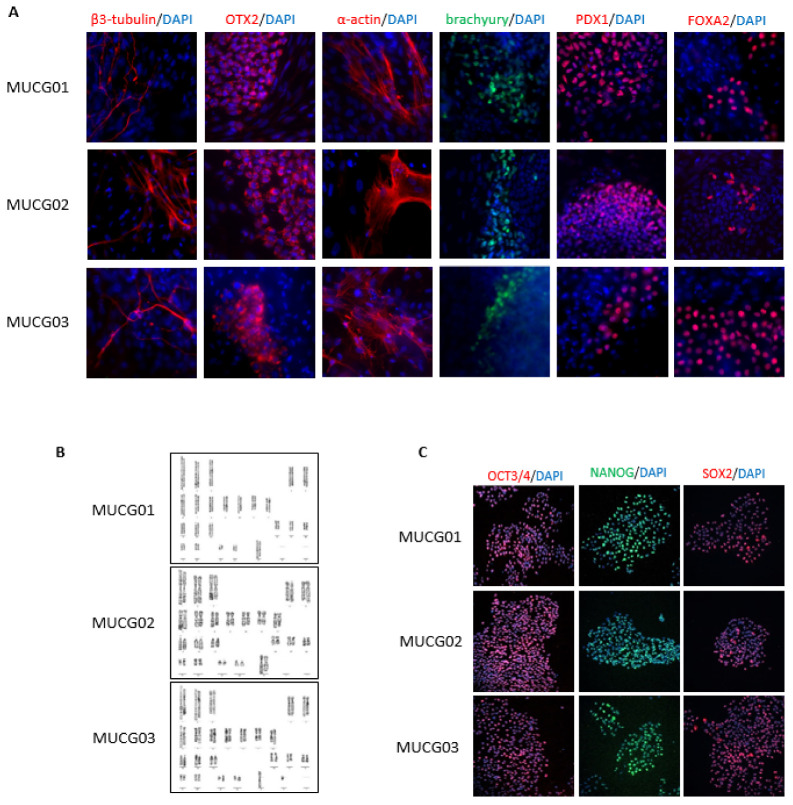
(**A**) Differentiation markers for the endoderm (FOXA2, PDX1), mesoderm (α-actin, brachyury), and ectoderm (β3 Tubulin, Otx2) were detected for the MUCG01, MUCG02, and MUCG03 clinical-grade hESC lines by immunocytochemistry. (**B**) Standard karyotypes without chromosomal aberration were detected for all hESC lines MUCG01: 46,XX; MUCG02: 46,XX; and MUCG03: 46,XY. (**C**) Pluripotency markers Oct3/4, Sox2, and Nanog were detected for the MUCG01, MUCG02, and MUCG03 clinical-grade hESC lines by immunocytochemistry.

The pluripotency of the clinical-grade hESC lines MUCG01, MUCG02, and MUCG03 was evaluated by immunocytochemistry and flow cytometry. Immunocytochemistry was performed during the expansion into master cell bank when all the hESC lines were positive for Oct3/4, Sox2, and Nanog markers (see Figure 3C). Flow cytometry was performed during the expansion into pre-master cell bank and again after thawing the working cell bank. The pluripotency markers were above the established limit of >70% positive cells for SSEA4, TRA-1-60, and TRA-1-81 markers at both timepoints, as shown in Table 4.

The differentiation potential of the clinical-grade hESC lines was confirmed by spontaneous differentiation. All the clinical-grade hESC lines MUCG01, MUCG02, and MUCG03 were positive for the endoderm (FOXA2, PDX1), mesoderm (α-actin, brachyury), and ectoderm (β3 Tubulin, Otx2) markers, as shown in Figure 3A.

The working cell bank was tested for after-thawing recovery by fulfilling the following criteria: >30 colonies were detected two days after thawing, >50% confluence and low differentiation was observed 5 days after thawing, and >60% viability was observed at the first after-thawing passage. 

### 2.4. Sterility

To ensure the safety of the personnel and the potential patients, the donors of embryos were tested for HIV 1/2, hepatitis B, hepatitis C, and syphilis, before gamete procurement with negative results (see Table 5) [30]. 

The sterility of the derived hESCs was tested during the first two passages after derivation, and then after the thawing of pre-master cell bank, master cell bank, and working cell bank with negative results for all three lines, MUCG01, MUCG02, and MUCG03. 

MUCG01, MUCG02, and MUCG03 tested negative for the presence of mycoplasma during the expansion into pre-master cell bank and after the thawing of the working cell bank. 

As there are no guidelines for the endotoxin limit for hESCs, we set it to 5 EU/mL for the working cell bank based on the current literature and the European Pharmacopoeia. The endotoxin levels for all three lines, MUCG01, MUCG02, and MUCG03, were under <1 EU/mL after the thawing of the working cell bank.

The environmental monitoring was set to detect the possible occurrence of bacteria or fungi in the clean rooms in order to prevent the contamination of the hESCs. No over the limit of bacteria or fungi was detected during the manufacture of the hESC lines MUCG01, MUCG02, and MUCG03.

### 2.5. Genetic Characterization

The identification of hESC lines was achieved by STR markers establishment. Seventeen STR loci, in addition to the sex-determining locus, Amelogenin, were detected by an external certified laboratory. All lines, MUCG01, MUCG02, and MUCG03, have a unique STR profile that was not found in the Cellosaurus database. The results are not published to protect the identity of the donors but are available from the author.

The HLA profile was established for MUCG01, MUCG02, and MUCG03, as shown in Table 6, in order to ensure the match of these cells and the potential patients that will be involved in cell therapies using clinical-grade hESC-derived cells.

No high-risk point mutation or intragenic deletion/insertion connected to cancer predisposition was detected in the investigated genes for the hESC lines MUCG01, MUCG02, and MUCG03 by the sequence panel CZECANCA [31].

Standard karyotypes without chromosomal aberration were detected for all hESC lines MUCG01: 46,XX; MUCG02: 46,XX; and MUCG03: 46,XY (see Figure 3). It is established practice to send cells alive for karyotyping, but the legislation in some countries, including the Czech Republic, prohibits the handling of hESCs without permission. Therefore, we implemented a fixing step prior to the transport. The fixed cells can be stored at 4 °C for 2 days, which provides handling space for transport.

## 3. Discussion

In this paper, we describe the cGMP system of the hESC manufacturing with the use of cGMP-defined xeno-free substrate, laminin 521, for hESC derivation and expansion together with the quality control requirements concerning sterility, safety, and characterization that was validated on three clinical-grade hESC lines, MUCG01, MUCG02, and MUCG03, registered at Human Pluripotent Stem Cell Registry (hPSCreg), for further clinical use.

Laminin 521 is a xeno-free defined surface that is suitable for hESC culture and is also used for the directed differentiation of hESCs into several cell types, for example, retinal pigment epithelial cells, hepatic cells, and dopaminergic neurons [2,3,4,27]. We used it as a derivation and culture substrate based on its availability in cGMP quality before the recently published reports, where the derivation of clinical-grade hESC lines on laminin 521 was described [32,33]. Our first experiments with laminin 521 confirmed its suitability for the derivation as we were able to derive and culture four research-grade hESC lines on this surface (data not shown). The concentration of laminin 521 and the adding of E-cadherin for the derivation was adjusted based on the published article of Rodin et al. [34]. 

Derivation and culture conditions are undeniably the main factors that have an impact on the quality of hESCs. We chose a hypoxic environment (5% O_2_) for both derivation and culture, because it has been proven that hypoxia supports the pluripotency of hESCs [29]. Moreover, the embryos that were used for the derivation were cultured under hypoxia as the ESHRE guidelines also recommend a low oxygen concentration for blastocyst culture [35].

The inner cell mass of an embryo can be accessed more easily when the zona pellucida is disturbed. It can be mechanically disturbed/removed during the derivation itself, but the collapse of the blastocyst cavity is, in our experience, almost inevitable. Therefore, the disruption was performed by laser to avoid the collapse of the blastocyst cavity (and animal-derived components) at the Center of Assisted Reproduction (CAR) University Hospital Brno embryological laboratory (Brno, Czech Republic) before the transport to CTEF. We hypothesize that the disruption of the *zona pellucida* by laser may influence the viability of blastocysts during the transport, and therefore the disruption of *zona pellucida* by laser in the clean rooms would be better. We suggest the development of a system for the evaluation of the post-transport viability of embryos when the *zona pellucida* is disrupted before transport in comparison with embryos whose *zona pellucida* is disrupted at the derivation site, as it could offer interesting results that could be beneficial for future derivations of clinical-grade hESCs. 

We were able to derive three clinical-grade hESC lines in the clean rooms of CTEF at St. Anne’s University Hospital Brno with a 7.9% success rate counting all 38 thawed blastocysts, even though some of them started to disintegrate after the thawing/transport. Mechanical derivation using micropipettes was performed in order to avoid the animal-derived substances (animal antibodies and guinea pig complement) that are involved in the immunosurgery of ICM [36]. Considering that success results of the hESC derivation for the mechanical isolation of inner cell mass combined with the laminin 521 surface have never been published, it is very difficult to make any comparison with other studies. However, there are papers where authors used human foreskin fibroblasts as a feeder and performed the mechanical isolation of inner cell mass in order to establish new hESC lines. Crook et al. achieved a 21% (four lines established, 19 embryos used) derivation efficiency and, with the same settings, Ström et al. achieved a 26% (five lines established, 19 embryos used) derivation efficiency [26,37]. Nevertheless, it is not clear if the total number of thawed embryos or the number of embryos that were selected for inner cell mass isolation were counted. Tannenbaum et al. reported that, from 100 embryos, 77 embryos survived the thawing, of which 34 developed into the blastocyst stage, from which 23 inner cell masses were isolated and plated onto irradiated cord WCB feeders [24]. This resulted in three hESC lines, which amounts to a 3% derivation efficiency if the total thawed embryos are counted. The efficiency of 7.9% (three lines established, 38 thawed embryos used) that we achieved might be affected by the thawing/transport conditions or laminin 521 surface, but the cause needs to be examined with more data.

The characterization strategy, safety testing, and specification criteria were based on the literature research and were in compliance with the Eudralex volume 4 [15]. The testing of adventitious viruses was planned not only for the donors but also for the final product. The derived clinical-grade hESC lines, MUCG01, MUCG02, and MUCG03, were tested for HIV1/2, HBC, HBV and syphilis before the release [30]. The testing of hereditary diseases is under consideration as it would add valuable information for further use in cell therapies. The whole genome sequencing could be also beneficial for some purposes, but the interpretation might be difficult [38]. 

The embryoid bodies assay was chosen for the confirmation of the germ layers’ formation. We considered this in vitro test sufficient and in line with the principles of the 3Rs (Replacement, Reduction, and Refinement); therefore, we see no need to use a teratoma assay that requires sacrificing mice [39]. Directed differentiation could also be beneficial, especially in cases where the facility knows about the use of clinical-grade hESCs and can confirm the differentiation into specific cell types, e.g., retinal pigment epithelium cells, dopaminergic neurons, or hepatic cells [2,3,4]. Our future plans include the differentiation of the MUCG01, MUCG02, and MUCG03 lines into retinal pigment epithelium cells for the cell therapy of age-related macular degeneration. Therefore, their potential to differentiate into ectoderm, tested by the embryoid bodies assay, is a crucial selection parameter for following the directed differentiation experiments. 

As the use of clinical-grade hESCs is increasing, there should be an emphasis placed on quality and safety. We suggest that other cGMP substrates and media be tested and the impact on the differentiation and pluripotency potential of hESCs evaluated. Finally, we find the establishment of international characterization and safety criteria for clinical-grade hESCs, together with specific characterization methods for hESC-derived cells used in cell therapies, most important [40]. 

## 4. Materials and Methods

### 4.1. Donor Testing

Both embryo donors were tested according to the CAR University Hospital Brno embryological laboratory (Brno, Czech Republic) procedures for the presence of HIV1/2, hepatitis B, hepatitis C, and syphilis with negative results.

### 4.2. Preparation and Transport of Embryos

Embryos were thawed using Warm Cleave or Warm Blast media (Vitrolife) at the CAR University Hospital Brno embryological laboratory (Brno, Czech Republic), a day or two days (according to frozen stages) before the transport. For lower embryo stages, the cultivation to the blastocyst stage in Blastocyst Medium (COOK) were performed. After reaching the blastocyst stage, the disruption of the *zona pellucida* was executed using a laser (OCTAX NaviLase). Prepared embryos in the hatched blastocyst stage were transferred to the Sydney IVF gamete buffer medium (COOK) and transported to the clean rooms of the Cell and Tissue Engineering Facility (CTEF, FNUSA-ICRC) using a temperature-controlled transport incubator at 37 °C (ICT-P portable incubator, Falc).

### 4.3. Derivation 

The derivation was performed immediately after receiving the embryos at CTEF. Each embryo was added by 100 µL pipette into the well of a 4-well dish (Thermofisher Scientific, San Jose, CA, USA) containing warm Sydney IVF gamete buffer medium on a stereomicroscope plate heated on 37 °C. Then, the embryo was transferred into a drop of the Sydney IVF gamete buffer medium (COOK) covered in the Sydney IVF culture oil (COOK) by denuding micropipette (cat. 005-300-A, Microtech IVF, Czech Republic). Each embryo was manipulated separately using biopsy (cat. 004-35-30A, Microtech IVF, Czech Republic) and holding micropipettes (cat. 001-120-30H, Microtech IVF, Czech Republic). The inner cell mass was sucked into the biopsy micropipette and placed in parallel with the holding micropipette with a small overlap to allow the inner cell mass to be mechanically biopsied with a fast swing (video published previously [41]). The inner cell mass was manipulated by denuding micropipette (cat. 005-150-C, Microtech IVF, Czech Republic) and placed into one well of a 4-well dish coated with 16.6 µg/mL (2.6 µg/cm^2^) Biolaminin 521 CTG (BioLamina) and 1.7 µg/mL (0.27 µg/cm^2^) E-cadherin (R&D Systems), and covered with a NutriStem^®^ hPSC XF Medium (Biological Industries, Beit-Haemek, Israel), containing 20 mg/mL human serum albumin (Vitrolife) and 10 µM ROCK inhibitor (Y27632, GMP, Bio-techne). The microscope plates remained heated at 37 °C during the entire derivation process.

### 4.4. Culture Conditions 

The derived hESCs were cultured under hypoxic culture conditions (5% O_2_, 5% CO_2_, 37 °C) in a NutriStem^®^ hPSC XF Medium (Biological Industries) with a daily medium change. The cells were passaged mechanically by an insulin syringe (B.Braun) and cultured on 16.6 µg/mL (1.9 µg/cm^2^) Biolaminin 521 CTG (BioLamina) with 1.7 µg/mL (0.27 µg/cm^2^) E-cadherin for the first three passages when colonies reached sufficient size. Then, the non-enzymatic passaging by 0.5mM EDTA was performed when the cells reached 70% confluence. The cells were exposed to 10 µM ROCK inhibitor (Y27632, GMP, Bio-techne) 1 h before and 24 h after passage. After an one-hour ROCK inhibitor treatment, the cells were rinsed with phosphate-buffered saline (PBS, Gibco), dissociated by 0.5mM EDTA (Invitrogen) in clumps, and placed on 10.0 µg/mL (1.1 µg/cm^2^) Biolaminin 521 CTG (BioLamina). The media change and cell culture evaluation were conducted by a trained staff member on a daily basis.

### 4.5. Freezing

The cells were rinsed with phosphate-buffered saline (PBS, Gibco), non-enzymatically passaged by 0.5 mM of EDTA (Invitrogen), and spined for 4 min/300 g. Cells (0.5 × 10^6^/mL) were resuspended in cold freezing medium consisting of 65% NutriStem^®^ hPSC XF Medium (Biological Industries), 25% CTS Knockout SR XenoFree Medium (Gibco), and 10% CryoSure-DMSO (WAK chemie) supplemented with 10 µM ROCK inhibitor and frozen in cryotubes (Nunc) placed in Corning CoolCell Freezing container at the rate of -1 °C/minute in −80°C freezer. Cryotubes were moved to nitrogen vapors (− 196 °C) after 24 h.

### 4.6. Mycoplasma Testing

The hESCs were allowed to grow until they reached 70% confluence, and then the medium change was stopped and, after two days, the medium was collected for testing. Samples were sent to Generi Biotech (Generi Biotech, Hradec Kralove, Czech Republic) where they were tested for mycoplasma by polymerase chain reaction (PCR).

### 4.7. Environmental and Personnel Monitoring

The daily environmental monitoring consisted of 2 settle plates (Tryptic Soy Agar-ICR 30mL, cat: 1460010120, Merck) placed in the laminar box, one settle plate in the B-grade room and another two settle plates in the C-grade room. The number of settle plates was set based on the risk analysis. 

Airborne particles were measured continuously in the laminar box (A-grade) and the B-grade room by build-in particle counters and in the C-grade room by two APC ErgoTouch Pro 2 particle counters. 

In addition to environmental monitoring, the monitoring of personnel was established by the finger dub imprints into plates (TSA with LTHTh-ICR 30mL, cat: 1460690120, Merck) after the manufacture on a daily basis and mask and gown imprints into plates (Tr. Soy Cont. A with LTHTh-ICR, cat: 1462310200, Merck) on a weekly basis.

### 4.8. Endotoxin Testing

The medium for the endotoxin testing was collected at 70% confluence of the culture. Samples were placed in −20 °C until they were sent to ITEST plus (Hradec Kralove, Czech Republic) for the endotoxin level establishment by the LAL test.

### 4.9. HLA Analysis

DNA was isolated using a QIAamp DNA blood mini kit (Qiagen). The HLA profile for Class I (HLA-A, -B, -C) and Class II (HLA-DR, -DQ) of the hESC lines was established by PCR-SSP by Department of Transfusion and Tissue Medicine, University Hospital Brno (Brno, Czech Republic).

### 4.10. After-Thawing Recovery

Cell attachment was examined 2 days after thawing by number of colonies. Growth was assessed by change in confluency between day 2 and 5 after thawing. The cells were counted, and the viability was measured during the first passage after thawing by cell counter Countess III.

### 4.11. Sterility

The sterility for the hESC lines during the culture and for the final product was tested in a culture medium after 24 h incubation with cells by the Laboratories of the Institute for Microbiology, St. Anne’s University Brno (Brno, Czech Republic), according to the Czech Pharmacopoeia 2.6.1.

### 4.12. Cancer Predisposition Sequencing

The cancer predisposition mutations for the hESC lines were established by CZECANCA sequencing panel targeting 219 cancer susceptibility genes. In addition to more than 50 clinically important high- and moderate-penetrance susceptibility genes, the panel also targets less common candidate genes with uncertain clinical relevance [31]. The following genes were involved: AIP; ALK; APC; APEX1; ATM; ATMIN; ATR; ATRIP; AURKA; AXIN1; BABAM1; BAP1; BARD1; BLM; BMPR1A; BRAP; BRCA1; BRCA2; BRCC3; BRE; BRIP1; BUB1B; C11orf30; C19orf40; casp8; CCND1; CDC73; CDH1; CDK4; CDKN1B; CDKN1C; CDKN2A; CEBPA; CEP57; CLSPN; CSNK1D; CSNK1E; CWF19L2; CYLD; DCLRE1C; DDB2; DHFR; DICER1; DIS3L2; DMBT1;DMC1; DNAJC21; DPYD; EGFR; EPCAM; EPHX1; ERCC1; ERCC2; ERCC3; ERCC4; ERCC5; ERCC6; ESR1; ESR2; EXO1; EXT1; EXT2; EYA2; EZH2; FAM175A; FAM175B; FAN1; FANCA; FANCB; FANCC; FANCD2; FANCE; FANCF; FANCG; FANCI; FANCL; FANCM; FBXW7; FH; FLCN; GADD45A; GATA2; GPC3; GRB7; HELQ; HNF1A; HOXB13; HRAS; HUS1; CHEK1; CHEK2; KAT5; KCNJ5; KIT; LIG1; LIG3; LIG4; LMO1; LRIG1; MAX; MCPH1; MDC1; MDM2; MDM4; MEN1; MET; MGMT; MLH1; MLH3; MMP8; MPL; MRE11A; MSH2; MSH3; MSH5; MSH6; MSR1; MUS81; MUTYH; NAT1; NBN; NCAM1; NELFB; NF1; NF2; NFKBIZ; NHEJ1; NSD1; OGG1; PALB2; PARP1; PCNA; PHB; PHOX2B; PIK3CG; PLA2G2A; PMS1; PMS2; POLB; POLD1; POLE; PPM1D; PREX2; PRF1; PRKAR1A; PRKDC; PTEN; PTCH1; PTTG2; RAD1; RAD17; RAD18; RAD23B; RAD50; RAD51; RAD51AP1; RAD51B; RAD51C; RAD51D; RAD52; RAD54B; RAD54L; RAD9A; RB1; RBBP8; RECQL; RECQL4; RECQL5; RET; RFC1; RFC2; RFC4; RHBDF2; RNF146; RNF168; RNF8; RPA1; RUNX1; SBDS; SDHA; SDHAF2; SDHB; SDHC; SDHD; SETBP1; SETX; SHPRH; SLX4; SMAD4; SMARCA4; SMARCB1; SMARCE1; STK11; SUFU; TCL1A; TELO2; TERF2; TERT; TLR2; TLR4; TMEM127; TOPBP1; TP53; TP53BP1; TSC1; TSC2; TSHR; UBE2A; UBE2B; UBE2I; UBE2V2; UBE4B; UIMC1; VHL; WRN; WT1; XPA; XPC; XRCC1; XRCC2; XRCC3; XRCC4; XRCC5; XRCC6; ZNF350; ZNF365. The sequencing and analysis were performed by Department of Cancer Epidemiology and Genetics, Masaryk Memorial Cancer Institute Brno (Brno, Czech Republic).

### 4.13. Flow Cytometry 

The collected cells were washed in phosphate-buffered saline (PBS, Gibco), resuspended in a PBS/EDTA (Invitrogen)/bovine serum albumin (BSA, Pan Biotech) solution, and incubated with antibodies for 10 min at 4 °C, after which the cells were rinsed with PBS, spun for 4 min/300 g, resuspended in PBS, and analyzed by a CytoFLEX flow cytometer (Beckman Coulter). The antibodies used were: human anti-TRA-1-60-PE, 1:75 (cat: 130-122-914, Miltenyi Biotec), human anti-SSEA-4-PE, 1:150 (cat: 130-122-914, Miltenyi Biotec), and human anti-TRA-1-81-APC, 1:90 (cat: 17-8883-42, Thermo Fisher Scientific, San Jose, CA, USA).

### 4.14. Cell Differentiation 

Differentiation to the three germ layers was supported by the medium: DMEM/F12 (Gibco), 15% knockout-serum replacement (Gibco), 1% non-essential amino acids (Sigma), 1% Glutamax (Gibco), 1% ZellShield (Minerva Biolabs), and 0.2% 2-Mercaptoethanol (Gibco). The cells were passaged in a low attachment 96-well dish (20 × 10^3^ cells/well) and cultured for 1 week until embryoid bodies were formed. The embryoid bodies were then transferred to 4-well dishes coated with 10.0 µg/mL Biolaminin 521 CTG (BioLamina), where they were allowed to attach and culture for another 14 days. 

### 4.15. Immunocytochemistry 

The cells were fixed with cold 4% paraformaldehyde (Sigma) for 20 min, permeabilized with 0.2% Triton X (Sigma) for 30 min, and blocked in 2.5% BSA (Pan Biotech) in PBS (Gibco) with 0.1% Tween 20 (Sigma) for 1 h. The fixed cells were treated with primary antibodies overnight at 4 °C. The primary antibodies used were: mouse anti-Oct3/4, 1:200 (cat: sc-5279, Santa Cruz Biotechnology, Dallas, TX, USA), rabbit anti-Nanog, 1:200 (cat: 4903, Cell Signaling Technology), mouse anti-Sox2, 1:100 (cat: MAB2018, R&D Systems), mouse anti-α-actin, 1:200 (cat: sc-130616, Santa Cruz Biotechnology), goat anti-FOXA2, 1:200 (cat: AF2400, R&D Systems), mouse anti-β3 Tubulin, 1:200 (cat: sc-850005, Santa Cruz Biotechnology), goat anti-PDX1, 1:17 (cat: AF2419, R&D Systems), rabbit anti-brachyury, 1:200 (cat: sc-20109, Santa Cruz Biotechnology), mouse anti-Otx2, 1:200 (cat: sc-514195, Santa Cruz Biotechnology). Then, secondary antibodies were added for 1 h. The secondary antibodies used were: anti-mouse Alexa 555, 1:500 (cat: 4409, Cell Signaling Technology), anti-rabbit Alexa 488, 1:500 (cat: 4412, Cell Signaling Technology, Danvers, MA, USA), donkey anti-goat NL557, 1:500 (cat: 4412, Cell Signaling). The stained cells were treated with 1 µg/mL DAPI (4′,6-diamidino-2-phenylindole) and observed under a fluorescence microscope. Acquiarium software (v2012-06-12, Faculty of Informatics, Masaryk University) was used to acquire the images. 

### 4.16. Karyotyping 

The cells were mitotically arrested in their logarithmic phase by adding 0.4 μg/mL KaryoMAX™ Colcemid™ Solution (Thermofisher) and subsequently incubated for 2 h under standard culture conditions (5% CO_2_, 37 °C). Following the detachment of cells with TrypLe express and a 25 min treatment with the hypotonic solution (DMEM/F12 with demineralized water in ratio 1:3), the cells were fixed with 4 °C methanol and acetic acid (3:1). A karyotype analysis was performed by the Cytogenetic Laboratory Brno (Brno, Czech Republic) with Giemsa-banding and microscopic examination. At least 40 metaphase spreads/samples were analyzed at a resolution of 450–500 bands/haploid set. 

### 4.17. STR Analysis 

DNA was isolated using a QIAamp DNA blood mini kit (Qiagen). Seventeen STR loci, in addition to the sex-determining locus, Amelogenin, were amplified and assessed by Generi Biotech (Hradec Kralove, Czech Republic).

## 5. Conclusions

In conclusion, we established a cGMP-defined xeno-free and feeder-free system for the derivation, culture, and banking of clinical-grade hESC lines that are suitable for preclinical and clinical trials. Following quality control testing with criteria concerned with sterility, safety, and characterization according to cGMP ensures the clinical-grade quality of the hESC lines. 

The established procedures were validated on three clinical-grade hESC lines, MUCG01, MUCG02, and MUCG03, which were manufactured aseptically in clean rooms according to EMA cGMP guidelines. The clinical-grade hESC lines MUCG01, MUCG02, and MUCG03 have a high level of pluripotency markers, the capacity to differentiate into all three germ layers, standard karyotypes, and no detected mutations connected to cancer predisposition, which make them excellent starting material for preclinical testing and cell therapy.

## Figures and Tables

**Table 1:** Overview of the derivations of the clinical-grade hESC lines.

Derivation No.	Number of Embryos ^1^	Clinical-Grade hESC Lines
1	8	2
2	10	1
3	10	0
4	10	0
4	38	3

^1^ All transported embryos were involved even though some of them started to disintegrate after the thawing/transport.

**Table 2:** Overview of the embryo stage and derivation conditions for the hESC lines MUCG01, MUCG02, and MUCG03.

	Conditions
Embryo stage	MUCG01: hatching blastocyst
	MUCG02: blastocyst
	MUCG03: hatching blastocyst
Derivation conditions	37 °C, 5% CO_2_, and 5% O_2_
Derivation medium	NutriStem hPSC XF medium containing 20 mg/mL human serum albumin and 10 µM ROCK inhibitor
Derivation substrate	16.6 µg/mL (2.6 µg/cm^2^) Biolaminin 521 CTG and 1.7 µg/mL (0.27 µg/cm^2^) E-cadherin

**Table 3:** Overview of the culture conditions, medium, passage, and freezing for the hESC lines MUCG01, MUCG02, and MUCG03.

	Conditions
Culture conditions	37 °C, 5% CO_2_ and 5% O_2_
Culture medium	NutriStem hPSC XF Medium
Substrate	p0–p3: 16.6 µg/mL (1.9 µg/cm^2^) Biolaminin 521 CTG and 1.7 µg/mL (0.27 µg/cm^2^) E-cadherin >p3: 10.0 µg/mL (1.1 µg/cm^2^) Biolaminin 521 CTG
Passage	ROCK inhibitor 1 h before and 24 h after passage
	p0–p3: mechanical passage >p3: 0.5mM EDTA passage, 1:10
Freezing medium	65% NutriStem hPSC XF Medium, 25% CTS Knockout SR XenoFree Medium, 10% CryoSure-DMSO, and 10 µM ROCK inhibitor
Number of frozen cells	0.5 × 10^6^ cells/mL in cryotube

**Table 4:** Flow cytometry results for the hESC lines MUCG01, MUCG02, and MUCG03. PMCB, pre-master cell bank; WCB, working cell bank.

Title 1	Expansion into PMCB	WCB
MUCG01	100.00% SSEA4^+^	99.97% SSEA4^+^
	98.89% TRA-1-60^+^	93.37% TRA-1-60^+^
	96.75% TRA-1-81^+^	99.00% TRA-1-81^+^
MUCG02	100.00% SSEA4^+^	99.77% SSEA4^+^
	97.79% TRA-1-60^+^	94.10% TRA-1-60^+^
	93.47% TRA-1-81^+^	98.81% TRA-1-81^+^
MUCG03	100.00% SSEA4^+^	99.96% SSEA4^+^
	97.97% TRA-1-60^+^	77.13% TRA-1-60^+^
	90.90% TRA-1-81^+^	97.89% TRA-1-81^+^

**Table 5:** Overview of the sterility tests for the hESC lines MUCG01, MUCG02, and MUCG03. PMCB, pre-master cell bank; MCB, master cell bank; WCB, working cell bank.

Test	Stage	Result ^1^
HIV 1/2, hepatitis B, hepatitis C and syphilis	donor testing	negative
sterility	derivation, PMCB, MCB, WCB	negative
mycoplasma	derivation, WCB	negative
endotoxin	WCB	<1 EU/mL
environmental monitoring	during the manufacture	under the limit ^2^
personnel monitoring	during the manufacture	under the limit ^2^

^1^ For the hESC lines MUCG01, MUCG02, and MUCG03. ^2^ Based on EudraLex Volume 4 Guidelines on Good Manufacturing Practice specific to Advanced Therapy Medicinal Products.

**Table 6:** HLA profile of the hESC lines MUCG01, MUCG02, and MUCG03.

hESC Line	HLA Profile
MUCG01	A*02; B*15, *44; C*03, 07; DRB1*04, *16; DQB1*03, *05
MUCG02	A*02; *32; B*15, *40; C*02, 07; DRB1*04, *11; DQB1*03
MUCG03	A*01; *24; B*08, *58; C*07; DRB1*03, *08; DQB1*02, *04

## Data Availability

Data supporting the reported results can be found at Human Pluripotent Stem Cell Registry (hPSCreg). MUCG01: https://hpscreg.eu/cell-line/CTEFe001-A (accessed on 1 August 2022); MUCG02: https://hpscreg.eu/cell-line/CTEFe002-A (accessed on 1 August 2022); and MUCG03: https://hpscreg.eu/cell-line/CTEFe003-A (accessed on 1 August 2022).

## References

[1] Thomson J.A. (1998). Embryonic Stem Cell Lines Derived from Human Blastocysts. Science.

[2] Plaza Reyes A., Petrus-Reurer S., Antonsson L., Stenfelt S., Bartuma H., Panula S., Mader T., Douagi I., André H., Hovatta O. (2016). Xeno-Free and Defined Human Embryonic Stem Cell-Derived Retinal Pigment Epithelial Cells Functionally Integrate in a Large-Eyed Preclinical Model. Stem. Cell Rep..

[3] Kirkeby A., Nolbrant S., Tiklova K., Heuer A., Kee N., Cardoso T., Ottosson D.R., Lelos M.J., Rifes P., Dunnett S.B. (2017). Predictive Markers Guide Differentiation to Improve Graft Outcome in Clinical Translation of HESC-Based Therapy for Parkinson’s Disease. Cell Stem. Cell.

[4] Kanninen L.K., Harjumäki R., Peltoniemi P., Bogacheva M.S., Salmi T., Porola P., Niklander J., Smutný T., Urtti A., Yliperttula M.L. (2016). Laminin-511 and Laminin-521-Based Matrices for Efficient Hepatic Specification of Human Pluripotent Stem Cells. Biomaterials.

[5] Lo B., Parham L. (2009). Ethical Issues in Stem Cell Research. Endocr. Rev..

[6] Verginer L., Riccaboni M. (2021). Stem Cell Legislation and Its Impact on the Geographic Preferences of Stem Cell Researchers. Eurasian Bus Rev..

[7] Schwartz S.D., Regillo C.D., Lam B.L., Eliott D., Rosenfeld P.J., Gregori N.Z., Hubschman J.-P., Davis J.L., Heilwell G., Spirn M. (2015). Human Embryonic Stem Cell-Derived Retinal Pigment Epithelium in Patients with Age-Related Macular Degeneration and Stargardt’s Macular Dystrophy: Follow-up of Two Open-Label Phase 1/2 Studies. Lancet.

[8] Kashani A.H., Lebkowski J.S., Rahhal F.M., Avery R.L., Salehi-Had H., Dang W., Lin C.-M., Mitra D., Zhu D., Thomas B.B. (2018). A Bioengineered Retinal Pigment Epithelial Monolayer for Advanced, Dry Age-Related Macular Degeneration. Sci. Transl. Med..

[9] Mehat M.S., Sundaram V., Ripamonti C., Robson A.G., Smith A.J., Borooah S., Robinson M., Rosenthal A.N., Innes W., Weleber R.G. (2018). Transplantation of Human Embryonic Stem Cell-Derived Retinal Pigment Epithelial Cells in Macular Degeneration. Ophthalmology.

[10] Menasché P., Vanneaux V., Hagège A., Bel A., Cholley B., Parouchev A., Cacciapuoti I., Al-Daccak R., Benhamouda N., Blons H. (2018). Transplantation of Human Embryonic Stem Cell–Derived Cardiovascular Progenitors for Severe Ischemic Left Ventricular Dysfunction. J. Am. Coll. Cardiol..

[11] Kobold S., Guhr A., Mah N., Bultjer N., Seltmann S., Seiler Wulczyn A.E.M., Stacey G., Jie H., Liu W., Löser P. (2020). A Manually Curated Database on Clinical Studies Involving Cell Products Derived from Human Pluripotent Stem Cells. Stem. Cell Rep..

[12] Ilic D., Ogilvie C. (2022). Pluripotent Stem Cells in Clinical Setting—New Developments and Overview of Current Status. Stem. Cells.

[13] Ilic D., Devito L., Miere C., Codognotto S. (2015). Human Embryonic and Induced Pluripotent Stem Cells in Clinical Trials. Br. Med. Bull..

[14] Desgres M., Menasché P. (2019). Clinical Translation of Pluripotent Stem Cell Therapies: Challenges and Considerations. Cell Stem. Cell.

[15] EudraLex—Volume 4. https://health.ec.europa.eu/medicinal-products/eudralex/eudralex-volume-4_en.

[16] Carpenter M.K., Dunnett S.B., Björklund A. (2017). Chapter 6—Regulatory Considerations for Pluripotent Stem Cell Therapies. Progress in Brain Research.

[17] Tannenbaum S.E., Reubinoff B.E. (2022). Advances in HPSC Expansion towards Therapeutic Entities: A Review. Cell Prolif..

[18] De Sousa P.A., Downie J.M., Tye B.J., Bruce K., Dand P., Dhanjal S., Serhal P., Harper J., Turner M., Bateman M. (2016). Development and Production of Good Manufacturing Practice Grade Human Embryonic Stem Cell Lines as Source Material for Clinical Application. Stem Cell Res..

[19] Crook J.M., Stacey G.N., Ilic D. (2014). Setting Quality Standards for Stem Cell Banking, Research and Translation: The International Stem Cell Banking Initiative. Stem Cell Banking.

[20] Abranches E., Spyrou S., Ludwig T. (2020). GMP Banking of Human Pluripotent Stem Cells: A US and UK Perspective. Stem. Cell Res..

[21] Reubinoff B.E., Pera M.F., Fong C.Y., Trounson A., Bongso A. (2000). Embryonic Stem Cell Lines from Human Blastocysts: Somatic Differentiation in Vitro. Nat. Biotechnol..

[22] Pekkanen-Mattila M., Kerkelä E., Tanskanen J.M.A., Pietilä M., Pelto-Huikko M., Hyttinen J., Skottman H., Suuronen R., Aalto-Setälä K. (2009). Substantial Variation in the Cardiac Differentiation of Human Embryonic Stem Cell Lines Derived and Propagated under the Same Conditions—A Comparison of Multiple Cell Lines. Ann. Med..

[23] Skottman H., Mikkola M., Lundin K., Olsson C., Strömberg A., Tuuri T., Otonkoski T., Hovatta O., Lahesmaa R. (2005). Gene Expression Signatures of Seven Individual Human Embryonic Stem Cell Lines. Stem. Cells.

[24] Tannenbaum S.E., Turetsky T.T., Singer O., Aizenman E., Kirshberg S., Ilouz N., Gil Y., Berman-Zaken Y., Perlman T.S., Geva N. (2012). Derivation of Xeno-Free and GMP-Grade Human Embryonic Stem Cells—Platforms for Future Clinical Applications. PLoS ONE.

[25] Ye J., Bates N., Soteriou D., Grady L., Edmond C., Ross A., Kerby A., Lewis P.A., Adeniyi T., Wright R. (2017). High Quality Clinical Grade Human Embryonic Stem Cell Lines Derived from Fresh Discarded Embryos. Stem Cell Res. Ther..

[26] Crook J.M., Peura T.T., Kravets L., Bosman A.G., Buzzard J.J., Horne R., Hentze H., Dunn N.R., Zweigerdt R., Chua F. (2007). The Generation of Six Clinical-Grade Human Embryonic Stem Cell Lines. Cell Stem. Cell.

[27] Albalushi H., Kurek M., Karlsson L., Landreh L., Kjartansdóttir K.R., Söder O., Hovatta O., Stukenborg J.-B. Laminin 521 Stabilizes the Pluripotency Expression Pattern of Human Embryonic Stem Cells Initially Derived on Feeder Cells. https://www.hindawi.com/journals/sci/2018/7127042/.

[28] Närvä E., Pursiheimo J.-P., Laiho A., Rahkonen N., Emani M.R., Viitala M., Laurila K., Sahla R., Lund R., Lähdesmäki H. (2013). Continuous Hypoxic Culturing of Human Embryonic Stem Cells Enhances SSEA-3 and MYC Levels. PLoS ONE.

[29] Forristal C.E., Wright K.L., Hanley N.A., Oreffo R.O.C., Houghton F.D. (2010). Hypoxia Inducible Factors Regulate Pluripotency and Proliferation in Human Embryonic Stem Cells Cultured at Reduced Oxygen Tensions. Reproduction.

[30] European Commission (2006). Commission Directive 2006/17/EC of 8 February 2006 implementing Directive 2004/23/EC of the European Parliament and of the Council as regards certain technical requirements for the donation, procurement and testing of human tissues and cells. Off. J. Eur. Union.

[31] Soukupová J., Zemánková P., Kleiblová P., Janatová M., Kleibl Z. (2016). CZECANCA: CZEch CAncer paNel for Clinical Application—Design and Optimization of the Targeted Sequencing Panel for the Identification of Cancer Susceptibility in High-risk Individuals from the Czech Republic. Klin. Onkol..

[32] Main H., Hedenskog M., Acharya G., Hovatta O., Lanner F. (2020). Karolinska Institutet Human Embryonic Stem Cell Bank. Stem. Cell Res..

[33] Kawase E., Takada K., Nakatani R., Yamazaki S., Suemori H. (2021). Generation of Clinical-Grade Human Embryonic Stem Cell Line KthES11 According to Japanese Regulations. Stem. Cell Res..

[34] Rodin S., Antonsson L., Niaudet C., Simonson O.E., Salmela E., Hansson E.M., Domogatskaya A., Xiao Z., Damdimopoulou P., Sheikhi M. (2014). Clonal Culturing of Human Embryonic Stem Cells on Laminin-521/E-Cadherin Matrix in Defined and Xeno-Free Environment. Nat. Commun..

[35] De los Santos M.J., Apter S., Coticchio G., Debrock S., Lundin K., Plancha C.E., Prados F., Rienzi L., Verheyen G., ESHRE Guideline Group on Good Practice in IVF Labs (2016). Revised Guidelines for Good Practice in IVF Laboratories (2015)†. Hum. Reprod..

[36] Kim S.J., Lee J.E., Park J.H., Lee J.B., Kim J.M., Yoon B.S., Song J.M., Roh S.I., Kim C.G., Yoon H.S. (2005). Efficient Derivation of New Human Embryonic Stem Cell Lines. Mol. Cells.

[37] Strom S., Inzunza J., Grinnemo K.-H., Holmberg K., Matilainen E., Stromberg A.-M., Blennow E., Hovatta O. (2007). Mechanical Isolation of the Inner Cell Mass Is Effective in Derivation of New Human Embryonic Stem Cell Lines. Hum. Reprod..

[38] Merkle F.T., Ghosh S., Genovese G., Handsaker R.E., Kashin S., Meyer D., Karczewski K.J., O’Dushlaine C., Pato C., Pato M. (2022). Whole-Genome Analysis of Human Embryonic Stem Cells Enables Rational Line Selection Based on Genetic Variation. Cell Stem. Cell.

[39] Buta C., David R., Dressel R., Emgård M., Fuchs C., Gross U., Healy L., Hescheler J., Kolar R., Martin U. (2013). Reconsidering Pluripotency Tests: Do We Still Need Teratoma Assays?. Stem. Cell Res..

[40] Karanu F., Ott L., Webster D.A., Stehno-Bittel L. (2020). Improved Harmonization of Critical Characterization Assays across Cell Therapies. Regen. Med..

[41] Souralova T., Holubcova Z., Kyjovska D., Hampl A., Koutna I. (2021). Xeno- and Feeder-Free Derivation of Two Sex-Discordant Sibling Lines of Human Embryonic Stem Cells. Stem Cell Res..

